# Whole-Genome Comparison Reveals Divergent IR Borders and Mutation Hotspots in Chloroplast Genomes of Herbaceous Bamboos (Bambusoideae: Olyreae)

**DOI:** 10.3390/molecules23071537

**Published:** 2018-06-26

**Authors:** Wencai Wang, Siyun Chen, Xianzhi Zhang

**Affiliations:** 1 Institute of Clinical Pharmacology, Guangzhou University of Chinese Medicine, Guangzhou 510006, China; wencaiwang@gzucm.edu.cn; 2 Germplasm Bank of Wild Species, Kunming Institute of Botany, Chinese Academy of Sciences, Kunming 650201, China; chensiyun@mail.kib.ac.cn; 3 School of Life Sciences, Sun Yat-sen University, Guangzhou 510275, China

**Keywords:** chloroplast genome, divergent IR borders, mutation hotspots, phylogenomics, herbaceous bamboos

## Abstract

Herbaceous bamboos (Olyreae) are a separate lineage with idiosyncratic traits, e.g., unisexual flowers and annual or seasonal flowering lifestyle, in the grass family. To elucidate the evolution of herbaceous bamboos we produced two complete chloroplast (cp) genomes from two monotypic genera i.e., *Froesiochloa* and *Rehia* via the genome-skimming approach. The assembled *F. boutelouoides* and *R. nervata* cp genomes were 135,905 and 136,700 base-pair (bp), respectively. Further whole-genome comparative analyses revealed that the cp genes order was perfectly collinear, but the inverted repeats (IRs) borders, i.e., the junctions between IRs and single copy regions, were highly divergent in Olyreae. The IRs expansions/contractions occurred frequently in Olyreae, which have caused gene content and genome size variations, e.g., the copy number reduction of *rps19* and *trnH(GUG)* genes in *Froesiochloa*. Subsequent nucleotide mutation analyses uncovered a greatly heterogeneous divergence pattern among different cpDNA regions in Olyreae cp genomes. On average, non-coding loci evolved at a rate of circa 1.9 times faster than coding loci, from which 20 rapidly evolving loci were determined as potential genetic markers for further studies on Olyreae. In addition, the phylogenomic analyses from 67 grass plastomes strongly supported the phylogenetic positions of *Froesiochloa* and *Rehia* in the Olyreae.

## 1. Introduction

Herbaceous bamboos (Olyreae) are a distinct lineage in Bambusoideae (Poaceae), with short and weakly lignified shoots, simple vegetative branching, unisexual flowers, and annual or seasonal flowering patterns [1,2,3,4]. Olyreae consist of circa 22 genera and 124 species, mainly distributed in Central and South America, except for *Buergersiochloa bambusoides* Pilg. being endemic to New Guinea and *Olyra latifolia* L. likely expanding from the New World to Africa/Madagascar [1,4]. Most herbaceous bamboos are diploid, although polyploidy is also known, e.g., tetraploid *Sucrea maculate* Soderstr. [5,6]. It is necessary to pay attention to herbaceous bamboos for understanding the specific traits of woody bamboos in Poaceae [6], e.g., lignification of shoots and gregarious and semelparous flowering style [1,3]. Unfortunately, previous studies on Bambusoideae mainly focused on the woody bamboos, i.e., temperate woody bamboos (Arundinarieae) and tropical woody bamboos (Bambuseae) [7,8,9,10]. The phylogenetic relationships among genera of Olyreae, as well as within the species-rich genera e.g., *Olyra* and *Pariana*, remain elusive and controversial [1,11]. Until recently, there have been few genomic resources available for herbaceous bamboos [11,12], limiting further comparative genomic studies in this particular grass lineage.

The chloroplast (cp) genomes of flowering plants range from 19 [13] to 218 kilobase (kb) [14] in size and typically consist of a large single-copy (LSC) region and a small single copy (SSC) region, which are separated by a pair of inverted repeats (IRs) [15]. Generally, the cp genome structure is highly conserved as quadripartite organization. However, exceptions are always present, such as the pine cp genomes only containing one IR region [15]. The cp genome size variations [16], gene losses and/or additions [17], and microhomology-mediated rearrangements [18] have also been revealed in several plants. The IRs size can also be largely divergent at the family level, e.g., in Fabaceae [15]. IRs expansions or contractions occurred frequently during plant evolution [19,20]. Furthermore, the lateral gene transfer (LGT) between cp, mitochondrial (mt), and nuclear (nr) genomes were common during plant evolution, e.g., in Amborellaceae [21] and Apocynaceae [22]. Since cp genome structural variations may be accompanied by speciation over time, such changes likely provide evolutionary information [23]. 

Previous studies of Arundinarieae [10,24] and Bambuseae [25] have revealed a highly conserved cp genome structure in terms of gene content, genome size and linear gene order. Nevertheless, recent cp genome comparative analyses have found contrasting IR borders, i.e., different conjunction regions between IRs and LSC/SSC (single copy regions), among herbaceous and woody bamboos [9,26]. That is *ndhF* across the boundary region of IRb/SSC in herbaceous bamboos (*Cryptochloa strictiflora*) while pseudogene ψ*ndhH* in the IRb/SSC border of woody bamboos (*Arundinaria gigantean*, *Guadua angustifolia* and *Dendrocalamus latiflorus*) [9,26]. In the same way, pseudogene ψ*ndhF* locates at the IRa/SSC boundary region of *C. strictiflora* cp genome while *ndhH* in *A. gigantean*, *G. angustifolia* and *D. latiflorus* [9,26]. The IR contraction and gene pseudogenization have led to a small gene content and genome size in *C. strictiflora* [9]. However, only one Olyreaea species (*C. strictiflora*) were included in the above analyses [9,26]. Whether there are conserved IR borders within herbaceous bamboos is still unknown. The evolution of *C. strictiflora*-like IR type in the Olyreae also needs to be uncovered. In addition, recently Ma et al. [27] reported an LGT event of mitochondrial DNA (circa 2.7 kb) to the plastid genome in one Olyreae genera (*Pariana*). Including more herbaceous bamboos, Wysocki et al. [12] found this LGT event from mt to cp genome were also present in another Olyreae genus i.e., *Eremitis*. However, due to the scarcity of Olyreae cp genomes data whether the LGT of mitochondrial DNA to cp genome is common in other herbaceous bamboos is still in the dark. 

Because of the nature of predominantly nonrecombinant and generally uniparental inheritance [28], cpDNA sequences have been extensively used in plant molecular phylogenetics studies [29,30]. Evolutionary rates of non-coding (i.e., intergenic regions and introns) and coding (i.e., exons of protein-coding genes) regions can vary tremendously in a given taxonomic group [31]. Lack of phylogenetic information is one of the major problems resulting in incompletely resolved phylogenetic trees in woody bamboos [32]. During the past few years morphometrics [33], allozymic markers [34] and DNA fragments (plastid spacer *trnD-trnT* and nuclear ITS) [11] have been used for reconstructing the phylogeny of *Raddia* and its allies within Olyreae. Nevertheless, all the above data failed to obtain a highly resolved phylogenetic tree of Olyreae [11,33,34]. Increasing the number of cpDNA loci with considerable variation would help to reconstruct the phylogeny of herbaceous bamboos [11]. Therefore, it is valuable to develop cp genome-wide molecular markers to unveil the evolutionary history within Olyreae. 

To date, only 10 out of 124 species of herbaceous bamboos have sequenced cp genomes [12,27]. Clearly the increasing number of complete cp genome sequences from Olyreae would improve our understanding of the cp genome evolution in herbaceous bamboos [9,12,26]. Based on the cp genome data the genome-wide highly variable cpDNA loci can further be mined [24,25]. In this study, we sequenced and analyzed two complete cp genomes of Olyreae, representing two separate genera *Froesiochloa* and *Rehia*, using the genome-skimming approach [35,36]. Through comprehensive comparative genomic analyses we aimed to (1) reveal the divergence pattern of cp genome IR borders in Olyreae; (2) identify the rapidly evolving cpDNA loci for subsequent phylogenetic studies on Olyreae; (3) resolve the phylogenetic positions of *Froesiochloa* and *Rehia* in Bambusoideae.

## 2. Results

### 2.1. Genome Assembly and Features

About 4.5 gigabytes (Gb) of data including circa 20 million clean reads were produced via genome-skimming sequencing for each species. There were 432,450 and 425,590 paired-end reads that were further aligned for *F. boutelouoides* and *R. nervata*, respectively (Table 1). Upon de novo and reference-assisted assembly, we obtained complete cp genomes of the two herbaceous bamboos with high genome coverage (>300×) (Table 1). The sequences of the two cp genomes have been deposited in GenBank with accession numbers: MH277033–MH277034.

**Figure 1:**
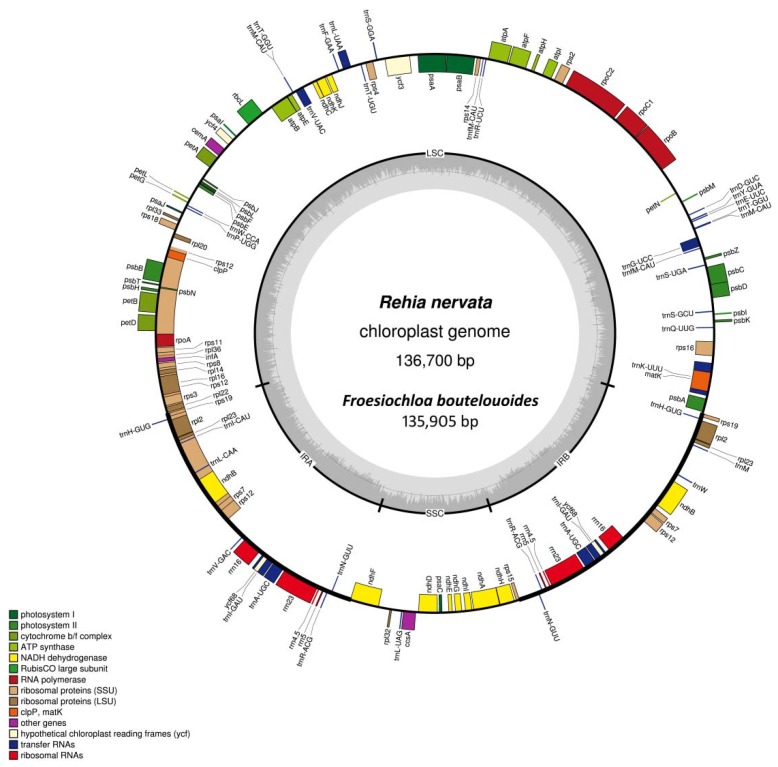
Chloroplast genome map of *Rehia nervata*. Genes belonging to different functional groups are color coded. Genes drawn outside the circle are transcribed clockwise and those inside are counterclockwise. The darker gray in the inner circle indicates the GC content of the chloroplast genome.

The cp genome size of *F. boutelouoides* and *R. nervata* was eventually determined as 135,905 and 136,700 base-pair (bp), respectively. Both the two cp genomes exhibited typical quadripartite organization of land plants, comprising a pair of IRs (19,993–21,248 bp) which separated one LSC region (80,931–82,935 bp) and one SSC region (12,984–13,273 bp) (Table 1, Figure 1). The comprehensive annotation analyses revealed that there were different numbers of protein-coding genes (79 vs. 84) and tRNA genes (37 vs. 38) in the two cp genomes (Table 1). Further detection revealed that the protein-coding genes *ndhF* and *ycf68* were likely pseudogenized in *F. boutelouoides*. Another three genes i.e., *rps12*, *rps19* and *trnH-GUG* lost one copy in *F. boutelouoides*. Eight intron-containing genes (*atpF*, *ndhA*, *ndhB*, *petB*, *petD*, *rpl2*, *rpl16*, *rps16*) had one intron each and only two genes (*rps12* and *ycf3*) contained two intervening introns.

**Figure 2:**
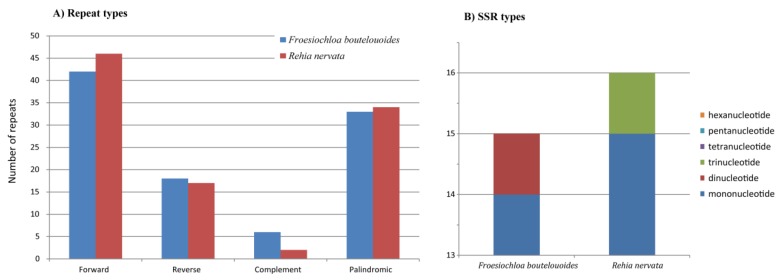
Genome repeat analyses. (**A**) Histogram showing the number of four types of repeat (forward, reverse, complement and palindromic) in the two Olyreae chloroplast genomes. (**B**) SSR unit size distribution in the two chloroplast genomes.

Four types of repeat (forward, reverse, complement and palindromic) and simple sequence repeats (SSRs) were investigated in the newly sequenced cp genomes (Figure 2). We found that forward repeats were the most abundant and palindromic repeats were secondarily abundant in both species (Figure 2A). In total, *F. boutelouoides* and *R. nervata* contained similar number of repeats. The analysis of occurrence and type of SSRs revealed that three kinds of SSRs, i.e., mono-, di-, and tri-nucleotide, were detected in the two cp genomes (Figure 2B). Mononucleotide repeats were the most dominant, up to 14 and 15 in *F. boutelouoides* and *R. nervata*, respectively. Only one dinucleotide SSR was found in *F. boutelouoides*, and only one trinucleotide SSR in *R. nervata* (Figure 2B). In all, 198 repeats and 31 SSRs were detected in cp genomes of these two herbaceous bamboos.

### 2.2. Whole Chloroplast Genome Comparison among Olyreae

**Figure 3:**
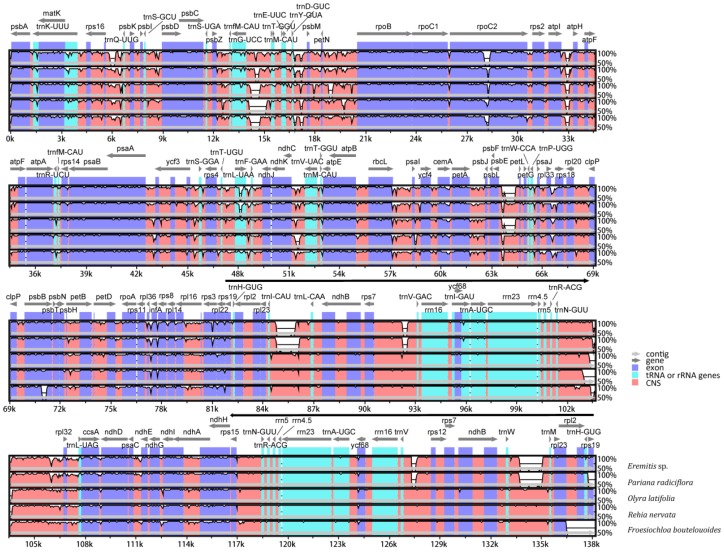
Whole chloroplast genome alignments for Olyreae. *Buergersiochloa bambusoides* was used as a reference and presented as the uppermost line in the VISTA-based identity plots. Thick arrowed black lines show the inverted repeat regions (IRs) in the chloroplast genome. All the chloroplast genome regions are color coded as protein coding, rRNA, tRNA or conserved non-coding sequences (CNS).

The two newly obtained cp genomes of Olyreae (*F. boutelouoides* and *R. nervata*) were compared with another four previously published ones (*B. bambusoides*, *Eremitis* sp., *P. radiciflora* and *O. latifolia*) that represented three subtribes of Olyreae i.e., Buergersiochloinae, Parianinae and Olyrinae (Table S1). Sequence differences among the six herbaceous bamboos were visually quantified by “percent identity plots” using *B. bambusoides* with annotation as a reference (Figure 3). Global alignments showed perfect synteny among the six cp genomes, suggesting that large-scale structural variations were not present in Olyreae cp genomes. Moreover, we found that sequence divergences were not uniform but remarkably heterogeneous among different regions of the cp genomes (Figure 3). As expected, the coding regions are more conserved than non-coding regions, and IRs more conserved than LSC and SSC regions. Interestingly, one large insertion was detected in the intergenic spacer of *trnI(CAU)*-*trnL(CAA)* located in the IRs of Parianinae cp genomes (*Eremitis* sp. and *P. radiciflora*) (Figure 3). Further investigation showed that the insertion sequences were about 2.7 kb and had homology (>90% similarity and coverage) with *rps7-atp6* mitochondrial loci of *Ferrocalamus rimosivaginus*.

**Figure 4:**
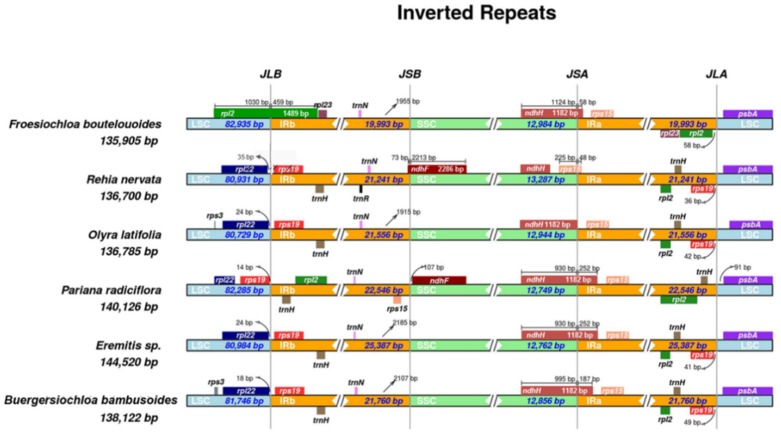
Comparison of IR boundaries among Olyreae chloroplast genomes. Each species and their corresponding chloroplast genome length are depicted to the left of each track. LSC: Large Single Copy region; SSC: Small Single Copy region; IRa/IRb: Inverted Repeat region a/b. JLB, JSB, JSA and JLA denote the junction sites of LSC/IRb, IRb/SSC, SSC/IRa and IRa/LSC, respectively. Genes drawn above/below the track indicate the direct/complement transcription.

Expansion and contraction of IR regions among the six representative Olyreae cp genomes were analyzed and the result is shown in Figure 4. We found that there were not constant IR borders in the herbaceous bamboos and the IR size varied from 19,993 bp to 25,387 bp. Complete *rpl22* and *rps19* genes resided in the LSC and IRb region, respectively, in *B. bambusoides*, *Eremitis* sp., *O. latifolia* and *R. nervata*; whereas in *F. boutelouoides* the *rpl2* gene straddled the LSC/IRb junction (JLB), and *rpl22* and *rps19* genes moved into the LSC region (Figure 4). The shift of *rps19* into the LSC region was also found in *P. radiciflora*. The *ndhF* gene may be pseudogenized due to frame shift mutations in all the six species except for *R. nervata* and *P. radiciflora* in which *ndhF* had intact opening read frame (ORF). In *R. nervata ndhF* straddled the IRb/SSC boundary (JSB) while in *P. radiciflora* this gene was completely located in the SSC region. The junction of SSC/IRa (JSA) fell into the *ndhH* gene in *B. bambusoides*, *Eremitis* sp., *P. radiciflora* and *F. boutelouoides*, but this gene was completely shifted into the SSC region in *R. nervata* and *O. latifolia*. The IR region of *F. boutelouoides* had contracted and caused the gene *rps19* to occur as single-copy gene within the LSC. Similarly, this phenomenon generated another single copy tRNA gene (*trnH(GUG*)), which further reduced the total number of plastid genes in *F. boutelouoides* (Table 1). Additionally, we also found that the distance from each boundary gene to the junction of IR and singe copy region (LSC or SSC) varied in different herbaceous bamboos (Figure 4). For example, the distance from *psbA* to IRa/LSC junction (JLA) ranged from 80 bp in *B. bambusoides* to 165 bp in *O. latifolia*.

### 2.3. Molecular Marker Development

**Figure 5:**
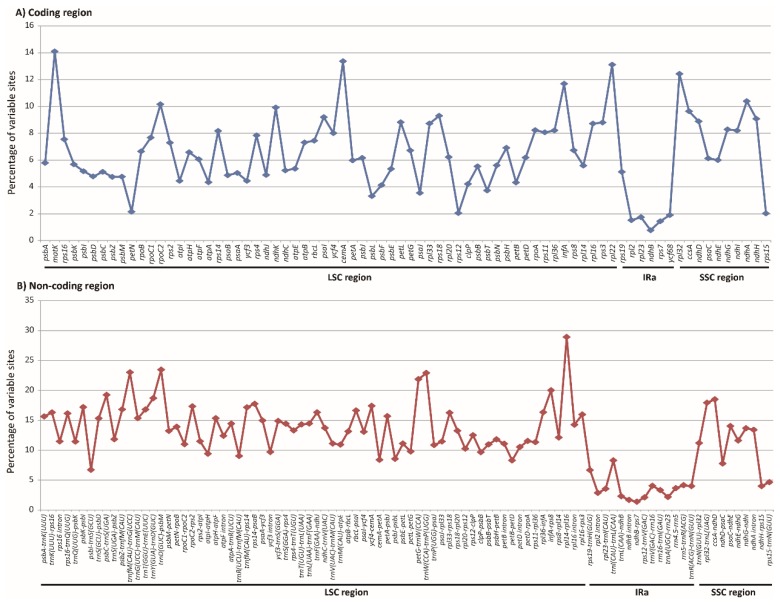
Percentage of variable characters (Single Nucleotide Polymorphisms, SNPs) in homologous loci among chloroplast genomes of Olyreae. (**A**) Coding region. (**B**) Non-coding region. The homologous loci are ordered according to their locations in the chloroplast genome.

All the available 13 Olyreae plastomes (Table S1) were analyzed to mine genome-wide molecular markers for subsequent evolutionary studies of herbaceous bamboos. The result showed that distribution patterns of variable characters, in terms of Single Nucleotide Polymorphisms (SNPs), differed greatly in plastid coding and non-coding loci with length >100 bp in Olyreae (Figure 5). The percentage of variable sites in non-coding loci ranged from 1.39% to 28.90% with mean value as 12.33% which was 1.9 times greater than that in the coding loci, 6.50% on average. The proportion of variability in most (69 out of 76) of the coding loci were less than 10% (Figure 5A). Only seven genes i.e., *cemA*, *infA*, *matK*, *ndhA*, *rpl22*, *rpl32* and *rpoC2* had their variations exceeded 10%, among which *matK* changed the most rapidly (14.10%). In contrast, there were 68 out of 94 non-coding DNA fragments showed relative high degree of divergence (>10.2%, Figure 5B).

Since it is convenient to design universal primers for PCR and sequencing experiments using the conserved coding sequences flanking the non-coding regions, 20 highly variable non-coding DNA fragments were chosen as potential molecular markers for subsequent evolutionary studies (Table 2). The proportion of variable characters (VCs) of these 20 non-coding loci all exceeded 13.3%, among which 13 had a percentage of VCs greater than 15.3% (Table 2). Furthermore, the percentage of parsimony informative characters (PICs) of the chosen 20 non-coding loci all exceeded 4.1%, with the intergenic spacer *trnD(GUC)-psbM* being the largest, up to 11.81% (Table 2).

### 2.4. Phylogenomic Inference

**Figure 6:**
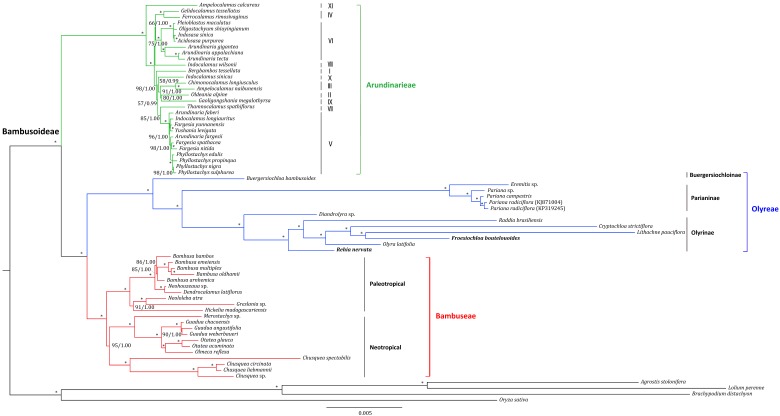
Congruent maximum likelihood (ML) and majority-rule consensus Bayesian inference (BI) tree for Bambusoideae based on the complete plastome sequences of 67 grasses. Values above the branches represent maximum likelihood bootstrap (MLBS)/Bayesian inference posterior probability (PP). * indicates nodes with 100/1.00 support values. The positions of newly sequenced herbaceous bamboos (*Froesiochloa boutelouoides* and *Rehia nervata*) were shown in bold.

A total of 67 grass plastomes including 63 Bambusoideae, three Pooideae and one Oryzoideae/Ehrhartoideae (Table S1) were used for phylogenomic inferences to uncover the evolutionary status of *Froesiochloa* and *Rehia*. The final aligned plastome matrix (full-plastome dataset) contained 109,051 unambiguous nucleotide characters. The two different methods i.e., maximum likelihood (ML) and Bayesian inference (BI) produced a congruent phylogenetic tree, shown in Figure 6. Bambusoideae and its three subordinate tribes (Arundinarieae, Bambuseae and Olyreae) were all highly supported as monophyly (100/1.00). A sister relationship of Bambuseae and Olyreae was revealed (100/1.00). In Arundinarieae, 11 proposed lineages (I to XI) were uncovered and the lineage XI (*Ampelocalamus calcareous*) was resolved as the basal most group (100/1.00). In Bambuseae, two lineages corresponding to paleotropical and neotropical woody bamboos were highly supported (100/1.00).

The Olyreae was divided into three subtribes (Buergersiochloinae, Parianinae and Olyrinae), each obtaining high statistic supports (100/1.00) (Figure 6). Buergersiochloinae diverged firstly, followed by Parianinae and Olyrinae (100/1.00). The monophyly of *Pariana* was highly supported in Parianinae (100/1.00). In Olyrinae, *Diandrolyra* sp. was supported as the basal most group (100/1.00). *R. nervata* was highly supported to be sister to a clade consisting of *Raddia brasiliensis*, *O. latifolia*, *Cryptochloa strictiflora*, *F. boutelouoides* and *Lithachne pauciflora*. Furthermore, the sister relationship of *F. boutelouoides* and *L. pauciflora* was strongly supported (100/1.00).

**Figure 7:**
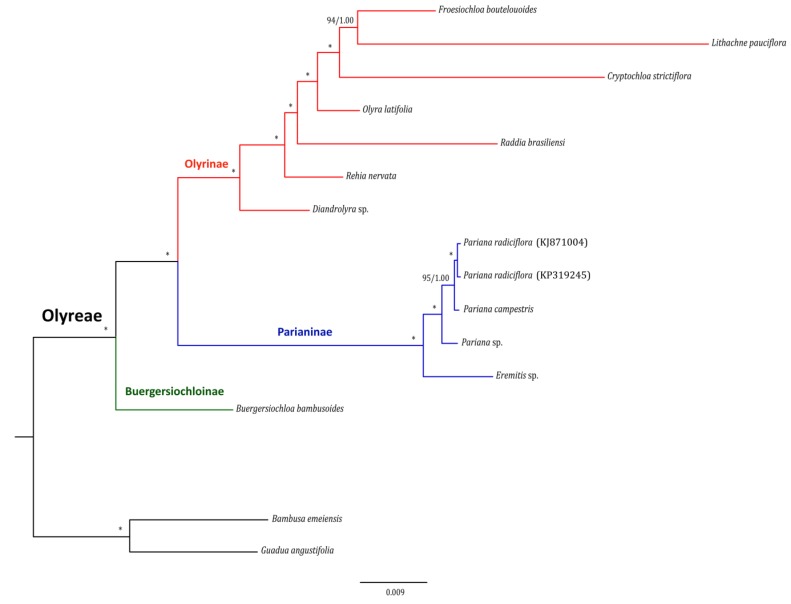
Congruent maximum likelihood (ML) and majority-rule consensus Bayesian inference (BI) tree for Olyreae based on the identified 20 highly variable cpDNA makers. Values above the branches represent maximum likelihood bootstrap (MLBS)/Bayesian inference posterior probability (PP). * indicates nodes with 100/1.00 support values.

To confirm the potential of the selected 20 Olyreae polymorphic loci (Table 2) in resolving the phylogeny of herbaceous bamboos, ML and BI analyses were conducted based on 13 Olyreae taxa and two woody bamboos as outgroups (*Bambusa emeiensis* and *Guadua angustifolia*) (Table S1). We found that the concatenated matrix of the identified 20 divergent loci (20-pastid-loci dataset) also generated a clearly resolved tree of Olyreae (Figure 7). The sister relationship of Olyrinae and Parianinae was supported with high statistic values (100/1.00). The phylogenetic relationships among the sampled seven genera in Olyrinae and the four taxa in *Pariana* were highly supported as well (Figure 7). Importantly, it is apparent that the topology of Olyreae derived from the 20-pastid-loci dataset (Figure 7) was congruent with the one resulted from the full-plastome dataset (Figure 6).

## 3. Discussion

### 3.1. Dynamic Chloroplast Genome IR Borders in Olyreae

Selective pressure may lead to chloroplast genome structure variations, for instance, the prominent cp genome reduction in non-photosynthetic plants, e.g., *Epipogium aphyllum* [13], *Orobanche* [37], and *Petrosavia stellaris* [38]. Complete sequencing and comparative analyses in this study revealed that cp genome size of *F. boutelouoides* (135.9 kb) is similar to that of *R. nervata* (136.7 kb) (Table 1), showing a typical cp genome size range (about 135 to 144 kb) of bamboos [12]. Whole cp genome alignments revealed a conserved organization and linear gene order between *Froesiochloa* (*F. boutelouoides*), *Rehia* (*R. nervata*) and another four representative cp genomes of Olyreae (Figure 3). This is consistent with the results of previous studies on temperate woody bamboos (Arundinarieae) [24], paleotropical [25] and neotropical [9] woody bamboos (Bambuseae). Nevertheless, in contrast to the woody bamboos, here we found divergent cp genome IR boundaries in the herbaceous bamboos (Figure 4). Even within the subtribe Olyrinae, three different types of IR borders were observed, *Froesiochloa*-, *Rehia*- and *Olyra*-like IR types (Figure 4). The *Rehia*-like IR type revealed here was similar to the *Cryptochloa*-like IR type unveiled previously [9,26]. Interestingly, this type of IR borders (*Cryptochloa/Rehia*-like) was also observed in grasses of the PACMAD clade (Poaceae) [39]. These observed diverse IR types in Olyreae strongly support the idea that IR borders evolved independently in Poaceae and did not obey the resolved phylogenetic relationships [40]. Moreover, due to the contraction of IRb into LSC, two genes (*rps19* and *trnH-GUG*) have become to be single-copy genes in *F. boutelouoides*. Similarly, the *ndhH* gene has shrunk into SSC completely in *R. nervata* and *O. latifolia* and the *rps15* gene has spread from IRa into SSC in *R. nervata*. The IRs contractions/expansions usually lead to cp gene content variations in plants [9,15], which may be one reason for the varied cp gene content in Olyreae.

It is noteworthy that a circa 2.7 kb insertion of mitochondrial DNA was uncovered in the *trnI(CAU)*-*trnL(CAA)* (*rpl23*-*ndhB*) region within the IR region of two Parianinae species (*P. radiciflora* and *Eremitis* sp.), not in Buergersiochloinae (*B. bambusoides*) and Olyrinae (*O. latifolia*, *R. nervata* and *F. boutelouoides*) (Figure 3). This result confirmed the recent finds of horizontal transfer of mitochondrial DNA to plastome in herbaceous bamboos [12,27]. Given the rarity of this type of intergenomic transfer in monocots [27], it is reasonable to hypothesize that the event of mitochondrial DNA insertion probably represents synapomorphy of the subtribe Parianinae. More species from Parianinae, especially representatives of the unsampled genus *Parianella*, will be sequenced to determine whether the lateral transfer of mitochondrial DNA is synapomorphy of the Parianinae.

Furthermore, two protein-coding genes (*ndhF* and *ycf68*) have lost intact ORFs because of frameshifting mutation to become pseudogenes in *F. boutelouoides* but hold good in *R. nervata* (Figure 1). These two genes seem to be intact in the SSC and IR regions, respectively, in woody bamboos [9,24,25]. The *ndhF* gene encodes 664 amino acids for chloroplast NADH dehydrogenase F subunit and is highly conserved in woody bamboos [24]. The function of hypothetical plastid gene *ycf68* is still not clear. The lack of essential plastid genes is not unusual in angiosperms, for example, several cp genes lost in certain groups, e.g., the *clpP* gene in *Camellia oleifera* [41] and *Primula poissonii* [42], the *accD* gene in *Trifolium* [43], the *rpl32* gene in *Populus* [44], and the *infA* gene in rosids [45]. We thus infer that the dysfunction of *ndhF* and *ycf68* in *F. boutelouoides* may imply that they have been functionally transferred into nuclear genome or replaced by some unknown genes. It is also possible for that *ycf68* per se appears to be nonfunctional given its position in the generally conserved IR regions [46].

### 3.2. Heterogeneous Sequence Divergence in Olyreae Chloroplast Genome

It has been revealed that sequence divergence was distinctly heterogeneous in several plant cp genomes, such as in Actinidiaceae [17] and in Eucommiaceae [47]. The alignment of six Olyreae cp genomes here also uncovered highly heterogeneous sequence divergences within this lineage (Figure 3). The IRs seem to be more conserved than the single-copy regions (LSC and SSC), consistent with the pattern revealed in woody bamboos [9,24]. This may be because homologous correction of two inverted repeat sequences could reduce the nucleotide variations of IR regions [48]. Additionally, the varied evolutionary rates among different cp genome regions suggest that partitioning strategy should be preferred in plastome phylogenomic practice for Olyreae. Several data-partitioning methods such as dividing cp genomes into LSC, SSC and IR regions [24], coding and non-doing regions [32], or independent genes [49,50] could be attempted.

Sequence variation analyses of each coding and non-coding loci with length > 100 bp across the 13 available Olyreae cp genomes show that each locus has distinct evolutionary pattern (Figure 5), just as observed in mimosoid legume [51] and Sileneae [52]. On the average, the sequence variations (in terms of SNPs) in Olyreae coding genes are about 2 times less than that in non-coding loci, which may be resulted from the strong selective pressure on the function constraint of coding sequences [53]. However, we cannot treat all things indiscriminately, for instance, four coding genes (*matK*, *cemA*, *rpl22* and *rpl32*) having their percentage of VCs greater than 12%. The accelerated variation rates have been observed for several plastid genes, such as *infA* and *rpl32* in *Eucommia* [47], *rps* in Saxifragales [54], and *rps* and *clpP* genes in *Silene* [55]. Relatively high level of mutation in *matK*, *cemA*, *rpl22* and *rpl32* genes in Olyreae may indicate that they are less constrained. Abnormal DNA replication, repair and/or recombination proposed for the speeded divergence of coding genes [51,56] seem to be the likely mechanism for this phenomenon. In addition, the high degree variation of *matK* may also imply its potential as a DNA barcode for herbaceous bamboos. 

Several chloroplast DNA fragments such as *ndhF*, *rbcL*, *rpl16*, *trnL-F* and *rpoC2* have been sequenced for a few herbaceous bamboos (*L. pauciflora*, *O. latifolia*, *P. radiciflora*, etc.) in Poaceae [40,57] or Bambusoideae phylogenetic studies [4,58,59]. Recently, the plastid spacer *trnD-trnT* was used for reconstructing the phylogeny of Olyreae with 37 samples of herbaceous bamboos [11]. However, the resulted trees for Olyreae in previous studies [11,57], especially when took a large taxon sample, have not been resolved clearly. The difficulty primarily lies in the lack of phylogenetic information because of the low sequence divergence in previously used molecular markers [4,11]. Therefore, developing rapidly evolving genetic markers is needed for the phylogenetic study of Olyreae, particularly of the large genera *Olyra* and *Pariana*, which would help to understand the evolutionary history of this unique bamboo group. The average percentage of variability in non-coding loci is higher (12.33%) than that in the coding loci (6.50%) (Figure 5). Moreover, based on the adjacent conserved coding sequences it is convenient to design universal primers for PCR and Sanger sequencing of non-coding DNA fragments with length ranging from circa 300 bp to 1500 bp [19,60]. Hence, 20 highly variable non-coding cpDNA fragments (Table 2) were chosen as potential molecular markers for subsequent genetic studies of the herbaceous bamboos. Some of these loci such as *rpl32-trnL(UAG)*, *ycf4-cemA* and *trnT(UGU)-trnL(UAA)* have been used in phylogenetic studies of temperate woody bamboos with relatively robust solutions [2,61]. Additionally, 15 and 16 cpSSR loci were identified in *F. boutelouoides* and *R. nervata*, respectively (Figure 2), which can also be used as potential cpSSR markers in population genetics studies of herbaceous bamboos, just as revealed in tropical woody bamboos [25].

### 3.3. Chloroplast Phylogenomic Estimation of Bamboos

Chloroplast genome is generally inherited as a unit with no recombination, allowing tracing the evolutionary relationships among species from different taxonomy levels [28,35]. During past ten years, whole plastome sequences have been used to estimate the phylogeny of Bambusoideae [12,32,62,63]. The full plastome analyses in this study confirmed the monophyletic tribes (Arundinarieae, Bambuseae and Olyreae) identified in previous studies [4,12]. Notably, the plastome tree topology (Figure 6) supported paraphyly of woody bamboos, consistent with the results derived from plastid genes [2,58] and plastomes [9,12,64]. All the sampled 11 major lineages of Arundinarieae (I–XI) were highly supported and resolved as (XI, ((VIII(IV, VI)), (I((X(III(II, IX)))(V, VII))))), in agreement with previous findings [2,32,63]. In Bambuseae, two monophyletic lineages representing neotropical and paleotropical woody bamboos were retrieved and the relationships within each lineage were clearly resolved as previous phylogenetic analyses of plastome sequences [9,12,25].

The relationships derived from our phylogenomic analyses among three subtribes (Buergersiochloinae, Parianinae and Olyrinae) of Olyreae are well-supported as stated previously [4,12]. All the internodes within Olyreae got maximum statistic support (Figure 6), which can be partly attributed to relatively rapid nucleotide substitution rate caused by the short generation times of herbaceous bamboos [65]. The deep divergence of *B. bambusoides* likely implied an interesting historical biogeographic scenario, i.e., Old World origin followed by New World dispersal and radiation [26,66]. *F. boutelouoides* and *R. nervata* were both clustered within the Olyrinae, agreeing well with current taxonomy [1,3]. These two species both lack fimbriae at the apex of foliage leaf sheaths [1,5], which may be a synapomorphic trait supporting their relatively close relationship. The phylogenetic relationships among *Diandrolyra* sp., *R. brasiliensis*, *O. latifolia*, *C. strictiflora* and *L. pauciflora* revealed here were also supported in previous studies [11,12]. The relationships among the sampled four individuals of the genus *Pariana* (*Pariana* sp., *P. campestris*, *P. radiciflora* (KJ871004) and *P. radiciflora* (KP319245) were also highly resolved, implying an enormous potential of plastome phylogenomic practice at deep (e.g., tribe) and shallow taxonomic level (e.g., genus) of bamboos.

Interestingly, the phylogenetic analyses of Olyreae using the selected 20 highly variable loci (Table 2) also obtained a highly resolved tree (Figure 7), which is consistent with the results of Bambusoideae full-plastome dataset (Figure 6) and previous studies [4,12,64]. This further confirmed that these 20 cp genome-wide loci can provide plentiful genetic information for the reconstruction of the phylogeny of Oyreae. Especially, under the circumstances of large taxon sampling, e.g., [11,57], the identified 20 rapidly evolving cpDNA loci could be useful molecular markers to facilitate phylogenetic inference of herbaceous bamboos with affordable budget.

Nuclear genes are biparentally inherited and can offer additional evidence for plant phylogenetic reconstruction [67,68,69]. Recent analyses of single-copy nuclear genes have revealed broad putative hybridizations in temperate woody bamboos [6,70,71]. The Bambuseae-Olyreae sister relationship has also be largely debated recently [6,72]. To comprehensively understand the evolutionary history of herbaceous bamboos the inclusion of both cpDNA loci and single-copy nuclear genes should be attempted in the future.

## 4. Materials and Methods

### 4.1. Plant Materials and DNA Sequencing

Fresh healthy leaves of *F. boutelouoides* and *R. nervata* were collected from Kunming Institute of Botany, Chinese Academy of Sciences, in Kunming City, Yunnan Province, China in September 2015. After collection the leaves were quickly drying in silica gel until use.

The method of genome-skimming sequencing [35,36] was used to obtain high-copy genomic sequences, e.g., plastome. Briefly, total genomic DNA was extracted from the leaves of single individual per species using CTAB method [73]. Paired-end (PE) libraries with insert size 400–500 bp were constructed from randomly fragmented genomic DNA following standard Illumina protocols (Illumina Inc., San Diego, CA, USA). The libraries were then sequenced on the Illumina HiSeq 2000 platform at Novogene (Beijing, China) to generate PE 100 bp reads, amounting to circa 4 Gb data for each sample.

### 4.2. Genome Assembly, Annotation and Repeat Analysis

Raw PE reads were filtered by removing low quality reads (phred scores lower than 20 for more than 10% of their bases). The CLC Genomics Workbench v7.5 software (CLC Bio, Aarhus, Denmark) was then used to assemble the *F. boutelouoides* and *R. nervata* cp genomes based on the remaining clean data. Three steps were conducted successively: (1) Contigs with coverage < 50 and sequences length < 300 bp were discarded; (2) The remaining contigs were aligned to the available cp genome of *O. latifolia* (GenBank accession number KF515509) as a reference by BLAST program (http://blast.ncbi.nlm.nih.gov/) [74] with e-value < 10^−5^; (3) The aligned contigs (≥90% similarity and query coverage) were identified as cpDNA sequences and ordered in terms of the reference genome. Small gaps were finally filled using PE clean reads as conducted in previous studies [75,76].

Since different approaches may produce inconsistent annotations [77], three independent methods were applied to annotate the *F. boutelouoides* and *R. nervata* cp genomes. Firstly, the assembled cp genomes were annotated using DOGMA software [78]. Secondly, these two cp genomes were input to Geneious v10.0 software [79] and annotated by using the “Annotation Transfer” option with *O. latifolia* cp genome (KF515509) as the reference. Thirdly, a newly developed method GeSeq [80] was used to obtain the annotations. We then compared and integrated the results from DOGMA [78], Geneious v 10.0 [79] and GeSeq [80]. The start/stop codons and intron/exon boundaries were checked carefully and adjusted manually where necessary in the Geneious v10.0 [79]. Moreover, all the annotated protein-coding genes were translated one by one in the Geneious v10.0 [79]. tRNAscan-SE 1.21 [81] was further applied to confirm the tRNA genes. OGDRAW program [82] was subsequently used to draw the physical map of the annotated cp genomes.

Repeated sequences with motif length > 8 bp in the cp genomes were determined by applying the program REPuter [83]. Four types of repeats i.e., forward (direct), palindromic (inverted), reverse, and complement repeats were cataloged following the manual of REPuter [83]. Furthermore, SSRs were analyzed by MISA perl script (http://pgrc.ipk-gatersleben.de/misa/). The minimum repeat units for mono-, di-, tri-, tetra-, penta-, hexanucleotide SSRs were set as 10, 6, 5, 5, 5, 5, respectively [17].

### 4.3. Whole-Genome Comparison and Mutation Hotspot Identification

Chloroplast genomes of four additional Olyreae species, i.e., *B. bambusoides* (KJ871000), *Eremitis* sp. (KJ870992), *P. radiciflora* (KP319245) and *O. latifolia* (KF515509), was downloaded from GenBank database. Together with our newly obtained cp genomes of *F. boutelouoides* and *R. nervata*, these six cp genomes were aligned via MAFFT program [84]. Variations in gene orders and sequences were then visualized in VISTA viewer [85] with *B. bambusoides* (KJ871000) as a reference. Moreover, the expansion and contraction of the IR regions at junction sites was inspected and plotted using IRscope [86]. The IR borders were compared carefully among the above six herbaceous bamboos.

To identify rapidly evolving DNA markers for subsequent phylogenetic and population genetic studies of herbaceous bamboos, all the available 13 Olyreae cp genomes (Table S1) were analyzed. Both the coding (exon) and non-coding (intergenic spacer and intron) loci in each cp genome were extracted separately using the “Extract Sequences” option of Geneious v10.0 [79]. Then we aligned homologous loci individually, using the program MUSCLE [87] implemented in Geneious v10.0 [79] with default settings. Each obtained alignment was further checked, and adjustments were made manually where necessary. The number of variable sites, in terms of SNPs, in each locus was tallied, respectively in MEGA v5.2 [88]. The proportion of nucleotide substitutions, i.e., SNPs for each coding and non-coding locus was then calculated as (NS/L) × 100, where NS = the number of nucleotide substitutions, L = the aligned sequence length.

### 4.4. Phylogenomic Analysis

To determine the phylogenetic positions of *F. boutelouoides* and *R. nervata*, 67 grass plastomes representing 41 genera of Bambusoideae, three genera of Pooideae and one genus of Oryzoideae/Ehrhartoideae (Table S1), were included for phylogenomic analyses. *Agrostis stolonifera*, *Brachypodium distachyon*, *Lolium perenne* and *Oryza sativa* was defined as outgroups according to previous studies [24,89,90,91]. Full-length plastomes were aligned using MAFFT program [84] with manual adjustment where necessary. IRb, long gaps (>1000 bp) and unreliably aligned regions was omitted from the matrix (full-plastome dataset) [9,12,25].

Furthermore, to reveal the potential of the 20 identified variable loci (Table 2) for phylogenetic reconstruction of Olyreae, we also analyzed the combined dataset of these 20 cpDNA markers using 13 herbaceous bamboos and two outgroups (*B. emeiensis* and *G. angustifolia*) (Table S1). Each locus was aligned individually, using MUSCLE [87] in Geneious v10.0 [79], and then concatenated as a supermatrix (20-plastid-loci dataset). Gaps were not coded in this dataset.

PartitionFinder 2 [92] is a program for automatically choosing best-fit partitioning schemes for genome-wide phylogenetic analyses. Here we used this program to divide the full-plastome and 20-plastid-loci datasets, respectively. jModelTest2 [93] was then applied to determine the best fitting nucleotide substitution models for each subset. The selected partition scheme and corresponding models were used in all downstream phylogenetic analyses. The two datasets (full-plastome and 20-plastid-loci) were respectively analyzed using ML and BI approaches. ML searches were respectively implemented in RAxML-NG v0.5.0b [94] (https://github.com/amkozlov/raxml-ng/releases) and IQ-TREE v1.6.5 [95] for over 10 times. Branch support (MLBS) was evaluated by 1000 ultrafast bootstrap replications. BI analyses were performed in MrBayes 3.2.6 [96]. Two independent runs with four chains were carried out for 100,000,000 generations with a sampling every 1000 generations till convergence (the average standard deviation of split frequencies < 0.01). We excluded the first 25% of trees as “burn in” and combined the remaining trees to estimate majority-rule consensus tree and posterior probabilities (PP). 

## 5. Conclusions

In summary, in this study we generated two complete cp genomes of Olyreae from two independent genera (*Froesiochloa* and *Rehia*) using the genome-skimming approach. Based on comprehensive genome-wide comparison we found that the cp genomes within Olyreae were highly conserved in terms of linear gene orders but displayed divergent IR borders without phylogenetic signal, which further resulted in the cp gene content variations. Moreover, obviously heterogeneous sequence divergences were uncovered in different regions of the Olyreae cp genome. Twenty rapidly evolving cpDNA loci have been identified as potential molecular markers for subsequent phylogenetic and population genetic studies of the herbaceous bamboos. The phylogenetic positions of *Froesiochloa* and *Rehia* in the Olyrinae lineage were strongly supported as well based on the cp genomes data. The phylogenetic relationships among the members of Olyreae have been robustly resolved using either cp genome data or 20 genome-wide highly variable cpDNA markers, indicating the potential of cp phylogenomics for tackling phylogenies in intractable herbaceous bamboos.

## Figures and Tables

**Table 1:** Details of the chloroplast genome sequencing, assembly and annotation.

	*Froesiochloa boutelouoides*	*Rehia nervata*
Total paired-end reads	20,158,220	20,330,116
Aligned paired-end reads	432,450	425,590
Mean coverage	318.2	310.6
Size (bp)	135,905	136,700
LSC ^a^ length (bp)	82,935	80,931
SSC ^b^ length (bp)	12,984	13,273
IR ^c^ length (bp)	19,993	21,248
Number of genes (unique)	124 (110)	130 (112)
Protein-coding genes (unique)	79 (75)	84 (77)
tRNAs (unique)	37 (31)	38 (31)
rRNAs (unique)	8 (4)	8 (4)
GC content (%)	38.7%	38.8%
Coding regions (%)	41.1%	44.1%

^a^ Large Single Copy region; ^b^ Small Single Copy region; ^c^ Inverted Repeat region.

**Table 2:** The 20 highly variable chloroplast non-coding loci identified in herbaceous bamboos.

Region	Length (bp)	Aligned Length (bp)	No. VCs ^a^	Percentage of VCs	No. PICs ^b^	Percentage of PICs
*trnD(GUC)-psbM*	854–1064	1177	276	23.45	139	11.81
*rpl32-trnL(UAG)*	612–753	820	147	17.93	53	6.46
*ycf4-cemA*	408–457	477	83	17.40	27	5.66
*psbK-psbI*	385–400	413	71	17.19	30	7.26
*psbZ-trnfM(CAU)*	462–818	946	159	16.81	61	6.45
*trnT(GGU)-trnE(UUC)*	463–493	542	91	16.79	24	4.43
*rbcL-psaI*	1117–1219	1347	224	16.63	82	6.09
*trnF(GAA)-ndhJ*	442–604	631	103	16.32	36	5.71
*trnK(UUU)-rps16*	380–563	595	97	16.30	26	4.37
*rps16-trnQ*(UUG)	721–1091	1252	202	16.13	60	4.79
*petA-psbJ*	956–988	1065	167	15.68	56	5.26
*atpH-atpF*	421–456	476	73	15.34	15	3.15
*trnS(GCU)-psbD*	866–982	1077	165	15.32	60	5.57
*psaA-ycf3*	583–640	736	110	14.95	32	4.35
*ycf3-trnS(GGA)*	535–595	621	91	14.65	38	6.12
*trnT(UGU)-trnL(UAA)*	733–785	853	122	14.30	45	5.28
*rpl16 intron*	1046–1117	1150	164	14.26	52	4.52
*psaC-ndhE*	462–513	592	83	14.02	30	5.07
*ndhC-trnV(UAC)*	777–917	1036	142	13.71	50	4.83
*ndhA intron*	1002–1017	1067	143	13.40	44	4.12

^a^ Variable Characters; ^b^ Parsimony Informative Characters.

## Supplementary Materials

The following are available online http://www.mdpi.com/1420-3049/23/7/1537/s1. Table S1: Plastomes of Bambusoideae and allied grasses analyzed in this study.
