# Supplementary material for: Whole-Genome Comparison Reveals Divergent IR Borders and Mutation Hotspots in Chloroplast Genomes of Herbaceous Bamboos (Bambusoideae: Olyreae)
Source: Molecules. 2018 Jun 26;23(7):1537. doi: 10.3390/molecules23071537 (PMC6099781; doi:10.3390/molecules23071537)
Supplement: Supplementary file 1 [file molecules-23-01537-s001.pdf]

Table S1. Plastomes of Bambusoideae and allied grasses analyzed in this study.

Classification	Taxon	GenBank
Bambusoideae/Arundinarieae	*Ampelocalamus calcareous*	KJ496369
	Gelidocalamus tessellatus	NC_024719
	Ferrocalamus rimosivaginus	HQ337794
	Pleioblastus maculatus	NC_024723
	Oligostachyum shiuyingianum	NC_024722
	Indosasa sinica	NC_024721
	Acidosasa purpurea	HQ337793
	Arundinaria gigantea	NC_020341
	Arundinaria appalachiana	KC817462
	Arundinaria tecta	KC817463
	Indocalamus wilsonii	NC_024720
	Bergbambos tessellata	KJ522748
	Indocalamus sinicus	KJ531442
	Chimonocalamus longiusculus	NC_024714
	Ampelocalamus naibunensis	NC_030767
	Oldeania alpine	KJ531443
	Gaoligongshania megalothyrsa	NC_024718
	Thamnocalamus spathiflorus	NC_024724
	Arundinaria faberi	NC_024713
	Indocalamus longiauritus	NC_015803
	Fargesia yunnanensis	NC_024717
	Yushania levigata	NC_024725
	Arundinaria fargesii	NC_024712
	Fargesia spathacea	NC_024716
	Fargesia nitida	NC_024715
	Phyllostachys edulis	NC_015817
	Phyllostachys propinqua	NC_016699
	Phyllostachys nigra	NC_015826
	Phyllostachys sulphurea	NC_024669
Bambusoideae/Bambuseae	*Bambusa bambos*	NC_026957
	Bambusa emeiensis [c]	NC_015830
	Bambusa multiplex	NC_024668
	Bambusa oldhamii	NC_012927
	Bambusa arnhemica	KJ870989
	Neohouzeaua sp.	NC_026963
	Dendrocalamus latiflorus	NC_013088
	Neololeba atra	NC_026964
	Greslania sp.	NC_026961

	Hickelia madagascariensis	NC_026962
	Merostachys sp.	KT373815
	Guadua chacoensis	KT373814
	Guadua angustifolia [c]	KM365071
	Guadua weberbaueri	NC_026991
	Otatea glauca	KP319243
	Otatea acuminat	KJ871003
	Olmeca reflexa	NC_026965
	Chusquea spectabilis	NC_026959
	Chusquea circinata	KP319241
	Chusquea liebmannii	NC_026969
	Chusquea sp.	KP319242
Bambusoideae/Olyreae [b]	*Buergersiochloa bambusoides* [a]	KJ871000
	Eremitis sp. [a]	KJ870992
	Pariana sp.	KP319246
	Pariana campestris	KP319244
	Pariana radiciflora	KJ871004
	Pariana radiciflora [a]	KP319245
	Diandrolyra sp.	KJ870991
	Rehia nervata [a]	MH277034
	Raddia brasiliensis	KJ870998
	Olyra latifolia [a]	KF515509
	Cryptochloa strictiflora	JX235348
	Froesiochloa boutelouoides [a]	MH277033
	Lithachne pauciflora	KJ871002
Pooideae	*Brachypodium distachyon*	NC_011032
	Agrostis stolonifera	NC_008591
	Lolium perenne	NC_009950
Oryzoideae/Ehrhartoideae	*Oryza sativa*	NC_027678

[a] The six representative Olyreae species included in the whole chloroplast genome comparison analyses. [b] The 13 available plastomes of herbaceous bamboos used for molecular marker development. [c] The two woody bamboos used as outgroups in molecular phylogenetic analyses of Olyreae (20-plastid-loci dataset). The newly sequenced chloroplast genomes in the current study are shown in bold.
